# Neuropathic Pain and Ultrasonography: A Multiperspective Literature Evaluation

**DOI:** 10.3390/diagnostics11091705

**Published:** 2021-09-17

**Authors:** Daniele Coraci, Serena Vincenza Capobianco, Marcello Romano, Salvatore Calvaruso, Michele Vecchio, Silvia Giovannini, Claudia Loreti, Augusto Fusco, Stefano Masiero, Valter Santilli, Luca Padua

**Affiliations:** 1 Department of Neuroscience, Section of Rehabilitation, University of Padova, 35121 Padua, Italy; stef.masiero@unipd.it; 2 CTO “Andrea Alesini” Hospital, 00145 Rome, Italy; capobianco.serena@gmail.com; 3 Neurology Unit, Azienda Ospedaliera Ospedali Riuniti Villa Sofia Cervello, 90146 Palermo, Italy; mc.romano1958@gmail.com; 4 Italian Society of Physical and Rehabilitation Medicine, 00198 Rome, Italy; dot.calvaruso@gmail.com; 5 Department of Biomedical and Biotechnological Sciences, Section of Pharmacology, University of Catania, 95123 Catania, Italy; michele.vecchio@unict.it; 6 UOC Neuroriabilitazione ad Alta Intensità, Fondazione Policlinico Universitario A. Gemelli IRCCS, 00168 Rome, Italy; silvia.giovannini@policlinicogemelli.it (S.G.); claudia.loreti@gmail.com (C.L.); augusto.fusco@policlinicogmelli.it (A.F.); luca.padua@unicatt.it (L.P.); 7 Department of Anatomical and Histological Sciences, Legal Medicine and Orthopedics, “Sapienza” University of Rome, 00185 Rome, Italy; valter.santilli@uniroma1.it; 8 Department of Geriatrics and Orthopedics, Università Cattolica del Sacro Cuore, 00168 Rome, Italy

**Keywords:** pain, ultrasound, rehabilitation, graph-theory, diagnosis, treatment

## Abstract

Among the tools useful for the management of neuropathic pain, ultrasound presents several advantages, shown by the literature. We assessed the scientific production about neuropathic pain and ultrasound from different points of view: general topics, journal categories, geographical origin and lexical analysis. We searched papers on PubMed using the Medical Subject Headings “neuropathic pain” AND “ultrasound”. We collected data about the journals where the papers were published, the country of the affiliation of the first author. For the lexical analysis, we evaluated the presence of selected words in the papers, and we built a graph representing the connections among words and papers. The papers were focused on the use of ultrasound as a diagnostic tool and guide for the therapy, assessing its application in different diseases such as Morton’s neuroma and piriformis syndrome. The most represented journal category was anesthesia while the most common country the United States of America. The lexical analysis confirmed the importance of ultrasound for diagnosis of specific disease and treatment of pain. The described approaches provide a multiperspective evaluation of the literature and may support the interpretation of the information contained by the papers.

## 1. Introduction

**Figure 1:**
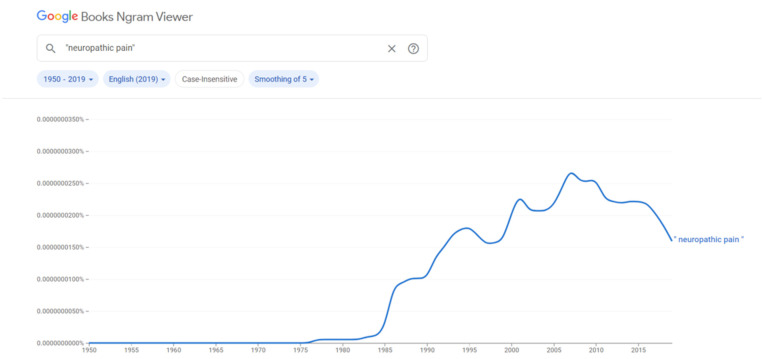
Presence of the term “neuropathic pain” in the books archived in Google. An increase is visible from the 1980s. In the *x*-axis the years are presented, in the *y*-axis the relative frequency of the terms in the digital sources.

Neuropathic pain (NeP) is a relatively common condition that affects the daily life of patients [1]. It can be caused by several diseases and may represent a challenge for clinical management. Indeed, the correct diagnosis of the symptom and the proper identification of the factors triggering or sustaining it are fundamental for the correct management. The impact of NeP is well documented in the literature [1]. In recent decades, the term “neuropathic pain” has shown a significant progression (Figure 1) [2].

The first challenge in the management of the NeP is related to the differentiation between NeP and the other types of pain, in particular nociceptive [3]. Proper knowledge of the features of the NeP is important because this allows the adequate formulation of questions to assess the symptom perceived by the patients. Specific questionnaires exist for this purpose, and they are able, in most cases, to identify the NeP [4]. In fact, it is characterized by specific feelings, peculiar localization, burn or electrical sensations, paresthesia, etc. [5]. However, the distinction between NeP and nociceptive pain is necessary, in particular because the treatment of the two conditions is different. In the case of nociceptive pain, the common painkillers may be enough, but in the case of NeP, a treatment based on drugs with central nervous system action is needed. This is due to the features of the NeP, in which an alteration of the afferent nervous system causes a sensitization able to reveal the pain even without a real damaging factor [3].

The second challenge in NeP management is related to the treatment choice, because the therapeutic options may have various efficacy and numerous side effects [6]. The possible drugs used for NeP include anticonvulsants, tricyclic antidepressant, selective serotonin reuptake inhibitors, and opioids. Recently, the use of nutraceuticals has been considered with acceptable effectiveness and low incidence of side effects [7].

Since the NeP is associated with many diseases, its impacts are not only related to the general quality of life of the patients, but also to the management itself. Indeed, the symptom may be extremely severe and influence the psychological status causing a lowering of the patient’s adherence to the treatment. Furthermore, the rehabilitation itself has shown an important reduction of its effectiveness when the NeP is present [8,9].

For these reasons, one of the most important targets in the management of the NeP concerns the identification of the primary reason for the symptoms. The number of diseases or clinical situations causing the NeP is very large and includes traumas, acquired of genetic neuropathies, drug-induced nervous diseases, radiculopathies, central nervous system diseases [3]. If the clinical evaluation allows a first diagnostic suspicion, the completion with specific tools may be useful to identify the alteration and to quantify its severity. Electrophysiological evaluation for the central and peripheral nervous systems may be considered in cases of NeP because these examinations can assess the whole nervous pathways and, hence, provide essential information for the diagnosis [10].

Considering the diagnostic tools, imaging techniques represent essential completion. These instruments can characterize from a morphological point of view, body parts. This can reveal the possible alteration in specific organs and can help in the identification of the conditions causing the NeP. Among these techniques, ultrasound (US) presents peculiar features, which make it the natural evolution of physicians’ hands and eyes [11]. US can see the structure in a completely safe way and in real time [12]. Furthermore, US allows a dynamic evaluation of the organs, differently from the common magnetic resonance. Besides this diagnostic usefulness, US is often used for the treatment [13,14]. The scientific literature constantly underlines the importance of US as a diagnostic tool and guide for therapy [15,16,17]. Considering the continuous increase of the literature production, the comprehension of the information conveyed by the papers may be difficult. In fact, the exemplification of the results is not always possible, with the risk of incomplete knowledge about a topic. Furthermore, by the usual literature review, we do not always examine the whole amount of information. Usually, we correctly focus on the clinical or epidemiologic information. Nevertheless, other data may be obtained with several following investigations. Through a wide and multiperspective analysis, we may discuss the scientific impact of a topic in the different world region. This should give the opportunity to analyze the possible reasons for this different impact. Additionally, the evaluation of the single words used to convey the meanings may reveal the homogeneity or not of the language used in scientific literature. These examples indicate some potentialities of the literature analysis that are often scarcely explored [18].

The aim of our paper was the assessment of the literature about the NeP and the US from multiple points of view. In particular, we considered: (1) the overall information about the literature concerning NeP and US; (2) the information about the journal categories and the geographical origins of the papers; (3) the lexical data to identify the relationships between the words and the published papers. With these approaches, we intended to summarize the current state of the art of the literature about NeP and US.

## 2. Materials and Methods

### 2.1. Literature Review

We searched on PubMed the papers about NeP and US using the following Medical Subject Headings (MeSH): “neuropathic pain” and “ultrasonography”. The two MeSH terms were linked by the Boolean operator “AND”, to collect the papers that included both terms. We just used the following filters: date (papers published in the last 10 years) and language (English). The search was performed on 24 February 2021. The titles and the abstracts of the found papers were exported as a text file using the specific PubMed function. After this initial collection, we excluded the non-pertinent papers based on the titles and the abstracts. The papers were considered non-pertinent when they concerned about non-human subjects or when NeP or US were not indicated as subjects of the study in the abstracts.

For each paper, using the titles and the abstracts, we evaluated: the main topic of each paper (diagnosis or treatment), the year of publication, the main disease of the study and the relative outcome measures.

### 2.2. Journal Categories and Geographical Origins

After the exclusion of non-pertinent papers, we have considered the journal in which each paper was published. We associated with this journal its main category as visible in Journal of Citation Reports. For each category, we calculated the total number of papers included and then we considered the percentage of recurrence of the category with respect to the total number of the papers.

For the assessment of the geographical origin of each paper, we considered the affiliation of the first author. In the case of multiple affiliations, just the first one was considered. For each country, the percentages with respect to the total number of papers were calculated and represented as a world map [18].

### 2.3. Lexical Network Based on Graph-Theory

The lexical network based on graph-theory (LENGTH) is an approach previously published that visualizes and analyzes the relationships of the words contained in the assessed papers. For the LEGTH, we imported the text file obtained from PubMed, after the exclusion of the non-pertinent papers, in the freeware software TXM 0.8.0 (Copyright © 2021–2018 ENS de Lyon, University of Franche-Comté, CNRS, Lyon, France) [19]. This allowed the calculation of the frequency of the words in titles and abstracts. In this way, we considered the 30 most common words in the text for further analysis. We calculated in which papers (titles and abstracts) the selected words were contained [18,20,21]. We built a matrix with values 0 (absence of the word in the paper) or 1 (every time the word was present). The matrix was imported into the freeware software Gephi 0.9.2 (licensed under CDDL and GNU GPL 3) [22]. This can transform the matrix in a graph, which is a mathematical object showing the interactions (edges) among different elements (nodes).

**Figure 2:**
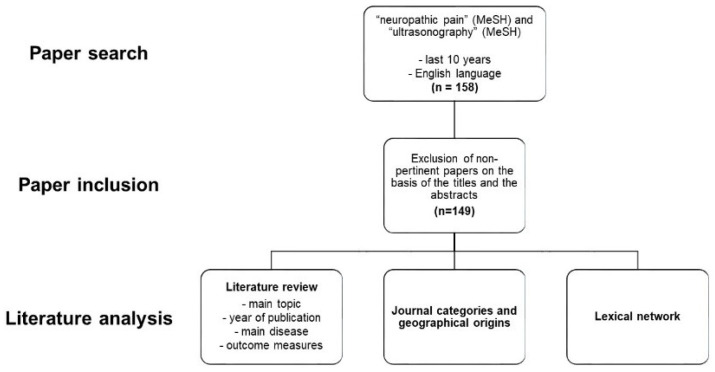
Flowchart of the literature analysis.

In our case, the nodes were the papers and the words, while the edges were the connections between them. The software can calculate specific parameters indicating the quantity and the quality of the connections. In particular, the degree indicates the number of the connections, which represents, for the words, their frequency. The modularity class shows the aggregation of nodes and, in our case, which words are “close” to others. The closeness centrality and the betweenness centrality indicate how well the nodes are connected with the others [18] (Figure 2).

## 3. Results

### 3.1. Literature Review

We found 158 papers on PubMed using the MeSH terms and the filters. Nine of these papers were excluded because not considered relevant for the aim of the study. Hence, the final number of the selected papers was 149. Among these, we found 57.05% of papers mainly focused on treatment, while 42.28% were focused on diagnosis. The topic of one paper was equally related to diagnosis and treatment. The literature about NeP and US showed a continuous growth with the major recent increases in 2016 and 2019. Concerning the assessed diseases, Morton’s neuroma was the most frequent, present in 15.57% of the studies, followed by piriformis syndrome in 14.75% and herpes/postherpetic neuralgia in 11.48% (Table 1). The most common outcome measures were the pain Visual Analogic Scale (VAS) and the pain Numeric Rating Scale (NRS), present in almost half of the studies. In a lower percentage (12.33%), the nerve dimension was considered to be outcome measures.

US showed a high level of effectiveness in studying the body structures with high capability to depict alterations useful for the diagnosis [23]. In the papers about Morton’s neuroma, US was extensively evaluated as a diagnostic tool and the neuroma size was considered predictive for clinical evolution [24]. In piriformis syndrome, US seemed useful for the diagnosis, but its effectiveness was especially visible for the treatment guidance, with a significant reduction of the pain using painkiller injections [25]. US showed a high level of success to guide the nerve block and, in Morton’s neuroma, US seemed to reduce the risks and increase the effectiveness of the treatment [26]. Concerning other diseases, the diagnostic contribution of US was even assessed in sciatica, where it showed morphological changes of the nerve, with enlargement and higher stiffness in the impaired nerve [27,28]. In the cases of herpes/postherpetic neuralgia, US was mainly used to guide the treatment, improving painful symptoms [29]. Patients with chronic pain were successfully treated with US-guided block [30].

### 3.2. Journal Categories and Geographical Origins

**Figure 3:**
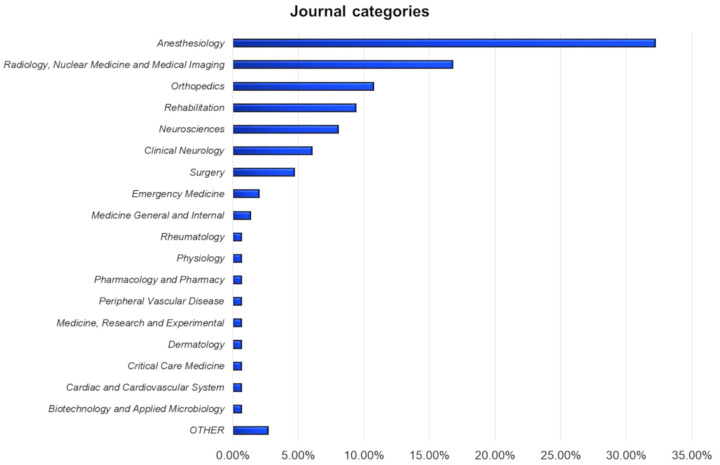
Journal categories of the papers. The results are expressed as percentage with respect to the total amount of papers.

The journal categories of the selected papers covered a wide range of medical fields (Figure 3). With 32.21%, “Anesthesiology” was the most represented, followed by “Radiology, Nuclear Medicine and Medical Imaging” with 16.78%. “Orthopedics” was the most represented surgical category (10.74%), while “Surgery” presented a very low frequency (4.70%). “Rehabilitation” was present just after “Orthopedics” and its percentage was higher than “Neuroscience” and “Clinical Neurology”. In 2.68%, we did not find any formal category.

**Figure 4:**
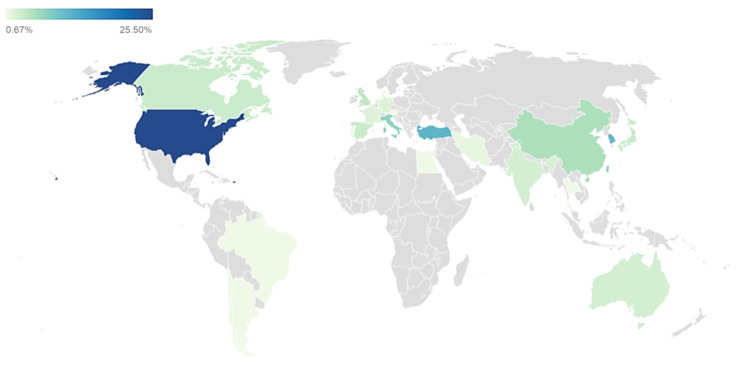
Geographical origin of the papers. The colors represent the percentage of the frequency of each country. The grey territories do not have any paper. The map was created by Datawrapper.

The geographical origin revealed the publications about NeP and US interest each continent (Figure 4). United States of America (25.50%) was the most common country, followed by Turkey ad Korea, respectively 11.41% and 10.74%. In Europe, the most represented countries were Italy (7.38%), United Kingdom (4.03%) and Spain (3.36%). In Africa, only the Arab Republic of Egypt showed publications, while in Southern America, three countries were present (Brazil, Argentina, and Chile). Finally, ten countries just presented one paper (0.67%) and, among them, the less populated was Luxembourg, while the most populated was Brazil.

### 3.3. Lexical Network Based on Graph-Theory

**Figure 5:**
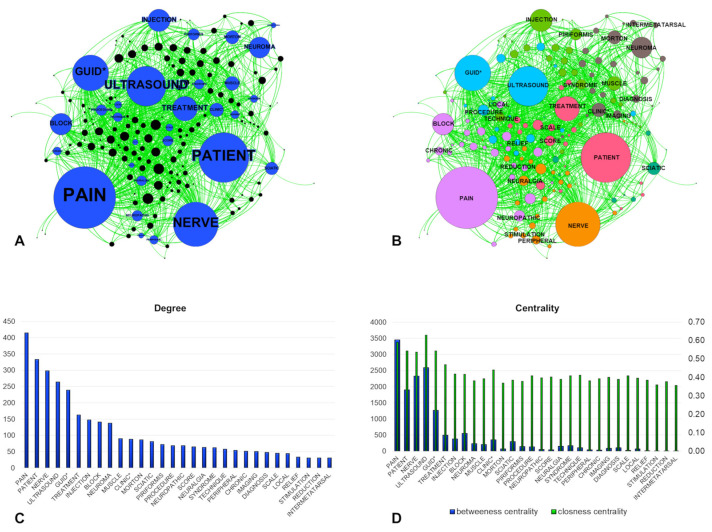
Results of the LENGTH approach. (**A**) The graph on the left represents the connections among words (blue) and papers (black), where the dimensions indicate the degrees. (**B**) The graph on the right considers the modularity classes indicated by different colors. The two bar charts show the degree (**C**) and the centrality values (**D**) of the words. The asterisk indicates the portion following the word root.

The LENGTH approach revealed PAIN was the most frequent word in the titles and abstracts (Figure 5). It was followed by the terms PATIENT, NERVE, ULTRASOUND and GUID *. The words directly related to the treatment were more common than the terms related to the diagnosis. Among the most cited words, rehabilitation did not appear. Regarding the diseases, NEUROMA and MORTON represented the most common words, followed by SCIATIC, PIRIFORMIS, and NEURALGIA.

The software can automatically locate the nodes to better visualize the associations and the modularity calculation supports the interpretation of node aggregation. Hence, we found seven node groups representing the major subtopics of the literature analysis. In particular, ULTRASOUND was associated with the terms related to the guiding procedures, while PAIN was associated with BLOCK and the terms defining the symptoms. The piriformis syndrome revealed an association with the injection, while Morton’s neuroma (and relative words) was especially connected with the diagnosis. Finally, PATIENT was related to the severity characterization of the symptoms and NERVE showed interactions with the peripheral nervous system and the nerve stimulation. SCIATIC appeared isolated from the other terms.

The centrality measures provided additional information about the words. Although ULTRASOUND was the fourth term for its frequency, it presented the highest level of connections in the graph, together with PAIN. The word DIAGNOSIS, while showed a relatively low degree, had higher centrality values compared to the words indicating the diseases, but lower than the words indicating the treatments. Similar behavior was visible for IMAGING. Considering the different syndromes, we found relatively high degrees, but the specific terms (MORTON and PIRIFORMIS) showed a low level of closeness centrality.

## 4. Discussion

The worldwide literature confirms the important contribution of US in management of the NeP. Many studies underline its positive impact in guiding the treatment, especially for the nerve block or the intramuscular injection [25,26,30]. As seen in journal categories, in recent years, some specific diseases of neurological, orthopedic, and rehabilitation interest (in particular Morton’s neuroma and piriformis syndrome) have been studied. Indeed, US application is fundamental to depict the morphological changes (especially in neuropathies) that support the diagnosis [27]. This is particularly evident in the case of Morton’s neuroma, where the nerve size is related to the outcome. In particular, a diameter of the neuroma larger than 6.3 mm predicted a failure of the steroid injection, with 74% sensitivity and 83% specificity. Additionally, Raouf and coworkers found the sensitivity of US in diagnosing the neuroma was about 93%. In this work, in about 57% of the cases, the neuroma was in the third space [31]. Furthermore, besides a similar diagnostic power in comparison with magnetic resonance, US is inexpensive, safe and able to dynamically assess body structures [24]. This important association between diagnosis and Morton’s neuroma is visible in the graph, where these two topics present the same modularity class. This should indicate the relevant interest of the diagnostic application in the disease. Additionally, the term IMAGING, showing the same modularity, likely sustains this interest.

In the management of the disease, US-guided injections appear more effective than blind procedures because, seeing the impaired organ to treat and the surrounding tissues, they limit the side effects, often related to the diffusion of the drugs in wrong sites [14,26].

In piriformis syndrome, Wu and colleagues assessed patients with clinical suspicion of piriformis syndrome with a low-frequency convex probe able to evaluate muscle and the sciatic nerve and with a high-frequency linear probe to study the nerve. They found piriformis muscle and sciatic nerve were enlarged and hypoechoic in the impairment side of the patients. Finally, the authors found a diagnostic sensitivity of around 70% for the muscle and nerve ad a very high specificity for the sciatic nerve [23]. In our analysis, US-guided injections seem mostly related to the piriformis syndrome, as visible in the graph [25]. For this disease, the local injections of anesthetic and corticosteroid should be considered when conservative approaches (based on oral drugs and physiotherapy) fail [25]. Furthermore, the US-guided block increases the precision of the injection targeting the drug close to the nerve [32]. For example, the clear visualization of the neuroma, with the probe positioned on the plantar surface, allows the guided injection inserting the needle from the dorsal surface [33]. The efficacy of the anesthetic block is particularly assessed by the literature and the graph visualizes this attention. In particular, the block is associated with the pain, with peculiar care to the chronic one. The same large frequency of the “Anesthesiology” journal category underlines the maximum attentiveness to this procedure. The second most common journal category, “Radiology, Nuclear Medicine and Medical Imaging”, may be related to diagnostic and treatment practice.

Interestingly, the term SCIATIC is apparently isolated, despite its relative common frequency in the selected papers. Its closeness centrality is relatively low. These data should indicate the specificity of most of the studies about sciatic impairment. This specificity is likely considered for the terms related to Morton’s neuroma. They appear relatively far from the other words and present a very low level of closeness centrality. This parameter is a measure of the distance of a node from the other ones. Hence, these speculations about the goodness of the connections of the node may be made. Obviously, they should be associated with the other parameters, in particular the word frequency.

The five most common terms in our analysis, which present the highest centrality values, include PAIN and ULTRASOUND. This is due to the MeSH terms used for the research. However, even if the MeSH term “neuropathic pain” was used, the word NEUROPATHIC just appears in the sixteenth position, considering its frequency, about 1:6 in comparison with PAIN. This is likely due to the omission of the adjective neuropathic in most of the times in which the pain is cited. However, among the five common words, PATIENT, NERVE and GUID* are present. This is interesting because the papers likely focus on clinical perspective and many of them consider the nerve impairment related to the neuropathy [34]. Finally, this ranking further underlines the importance of US to guide the treatment.

The geographical origin of the papers shows stimulating data. First, the number of papers, as expected, is not directly correlated with the population. The United States of America, as often visible, is a very prolific country, while China and especially India, the two most populated countries in the world, do not show a significant amount of published paper. This should be related to their relatively recent approach in the worldwide scientific literature. In our analysis, Turkey confirms its increasing impact, already visible in other scientific productions related to US. Interestingly, two small countries, Cyprus and Luxembourg, and a very large one, Brazil, showed one single paper. The results underline the dissociation between the population and the literature production, and they should be further studied considering, for each country, different points of view: culture, language, history, society, income, healthy policies, education systems and, specifically, relations with the virus diffusion [35].

In general, the LENGTH approach reveals the connections among words and papers, considering the lexical point of view of the papers. This method may reveal information enclosed in the texts and allow speculations about the way to convey specific messages by the words. The calculation of the parameters may be relevant because of the quantification of the position and the impact of a word in the network. Finally, the considerations should be always related to the “usual” revision process to support and translate them into useful information. The approach can be used for the visualization of the state of the art of literature, can support the consideration of the information contained in the papers and can help in identifying the topics to widely assess.

The approach presents some limitations. First, paper exclusion depends on the abstracts and the examiner’s experience. Second, the word selection itself depends on experience, because, despite the calculation of the frequencies, some words are not considered pertinent. Additionally, the words are analyzed in the titles and abstracts and not in the entire document, but this allows a simplification of the analysis without the restriction due to the unavailability of some papers.

## 5. Conclusions

In conclusion, the management of the NeP includes US application both for the diagnosis and for the treatment. For the diagnosis, specific diseases such as Morton’s neuroma and piriformis syndrome can be evaluated. For the therapy, US is a fundamental help in guiding the injection and in performing nerve blocks. The published papers mainly belong to journals of anesthesiology and radiology and cover countries of all continents. Finally, the lexical analysis confirms the US impact for diagnosis and treatment and reveals specific relationships with words indicating organs, diseases, techniques, and clinical components.

## Figures and Tables

**Table 1:** Presentation of the most frequent findings.

**Role**	Diagnosis and Treatment	0.67%
	Diagnosis	42.28%
	Treatment	57.05%
**Diseases**	occipital neuralgia	2.46%
	peripheral nerve disease	4.10%
	sciatic neuropathy	4.10%
	herpes/postherpetic neuralgia	11.48%
	piriformis syndrome	14.75%
	Morton’s neuroma	15.57%
	Other	47.54%
**Outcome Measures**	nerve dimension	12.33%
	pain NRS	16.44%
	pain VAS	26.03%
	Other	45.21%

## Data Availability

The graph generate by Google Ngram Viewer can be obtained through the following site: https://books.google.com/ngrams (accessed on 24 February 2021).
